# The portrayal and perceptions of cesarean section in Mexican media Facebook pages: a mixed-methods study

**DOI:** 10.1186/s12978-022-01351-8

**Published:** 2022-02-22

**Authors:** Martha Vazquez Corona, Ana Pilar Betrán, Meghan A. Bohren

**Affiliations:** 1 Gender and Women’s Health Unit, Centre for Health Equity, School of Population and Global Health, University of Melbourne, Carlton, VIC Australia; 2 UNDP/UNFPA/UNICEF/WHO/World Bank Special Programme of Research, Development and Research Training in Human Reproduction (HRP), Department of Sexual and Reproductive Health and Research, World Health Organization, 20 Avenue Appia, Geneva, Switzerland

**Keywords:** Maternal health, Cesarean section, Women’s health, Childbirth, Media analysis, Facebook analysis, Mexico

## Abstract

**Background:**

Mexico has one of the highest rates of cesarean sections globally at over 45%. There is limited research about social factors influencing these rates. This study explores the portrayal and perceptions of cesarean section in Facebook media pages to better understand the socio-cultural context of childbirth in Mexico.

**Methods:**

This is a mixed-methods social media analysis using two data sources. First, to study the portrayal of cesarean section, we identified ten Mexican media Facebook pages with the largest audiences (based on number of page “likes”). We searched these pages for articles containing the word “cesárea” (Spanish for cesarean section), and posts (articles) were eligible for inclusion if they contained the word “cesárea”. Second, to understand perceptions of cesarean section portrayal, we extracted comment threads of each Facebook post sharing the included articles. We performed a qualitative thematic analysis of articles and a quantitative content analysis of comments.

**Results:**

We included 133 Facebook posts depicting 80 unique articles and identified three major themes: (1) information about cesarean section, (2) inequality and violence against women, (3) governance failures. Cesarean section was portrayed as a lifesaving procedure when medical necessary, and riskier than vaginal birth, with a longer recovery time, and possible negative health consequences. We extracted comments from 133 Facebook posts, and 6350 comments were included. We inductively developed 20 codes to then classify comments under six major categories: (1) violence and discrimination, (2) health and health services, (3) mode of birth choice, (4) disbelief at information about cesarean section, (5) abortion, and (6) discontent at the government.

**Conclusions:**

We found that Facebook media did not promote cesarean section over vaginal birth, and risks and consequences were mostly represented reliably. Perceptions about the portrayal of cesarean section showed strong discontent and distrust against providers and the health system, as well as rejection of factual information about the consequences of cesarean section. We documented gross gender inequality and violence against women, highlighting the urgent need for human rights approaches to maternal health to address these inequalities and prevent harmful practices. Our study also contributes to the emerging field of social media analysis, and demonstrates clear areas where social media communication can be improved.

**Supplementary Information:**

The online version contains supplementary material available at 10.1186/s12978-022-01351-8.

## Plain Language Summary

Cesarean section is a medical intervention that can save women and babies when there are complications during pregnancy or birth. Mexico has one of the highest rates of cesarean section in the world (45%); much higher than what we would expect. We do not fully understand why this is happening, but we think social influences are important.

We know that traditional and social media are important social influences on health and health-seeking behaviors in other countries. In our study, we aimed to explore how cesarean section is portrayed in Facebook Mexican media pages, and how people (Facebook users) interacted with these articles. To do this, we identified the most popular Facebook media pages in Mexico.

Next, we searched for all articles posted to these pages. We found 80 articles and studied them to understand how they discussed cesarean section. We found that media articles posted on Facebook did not encourage women to have cesarean section, and they correctly showed risks and consequences.

Then, we explored the comments from Facebook users that were attached to these posts about cesarean section. We found 6350 comments, and classified each comment based on the what the Facebook users said.

Lastly, we connected the main themes of the articles to the types of comments. We found that Facebook users distrusted the Mexican health system, rejected information about the consequences of cesarean section, and often responded with the content with sexist and aggressive comments against women.

Our research shows that while there is accurate and useful information on social media about cesarean section, other social issues like gender inequality and violence may influence pregnancy and childbirth.

**Supplementary Information:**

The online version contains supplementary material available at 10.1186/s12978-022-01351-8.

## Resumen

**Antecedentes:**

México tiene una de las mayores tasas de cesáreas con más del 45%. Este estudio tiene como objetivo explorar la representación y percepción de la cesárea en las páginas de medios de comunicación en Facebook para comprender mejor el contexto sociocultural del parto en México.

**Métodos:**

Este es un análisis de redes sociales de métodos mixtos. Para estudiar la representación de la cesárea, identificamos diez páginas de Facebook de los medios de comunicación mexicanos con las mayores audiencias. Incluimos publicaciones de artículos de dichas páginas si contenían “cesárea” en el título, encabezado, o texto previo de la publicación. Después, para comprender las percepciones de la representación de la cesárea, extrajimos los comentarios de cada publicación incluida. Realizamos un análisis temático de los artículos y un análisis de contenido de los comentarios.

**Resultados:**

Incluimos 133 publicaciones de Facebook con 80 artículos únicos e identificamos tres temas principales: (1) información sobre la cesárea, (2) desigualdad y violencia contra las mujeres, (3) fallas de gobernanza. La cesárea se describió como un procedimiento que salva vidas cuando es médicamente necesario, más riesgoso que el parto vaginal, con un mayor tiempo de recuperación, y posibles consecuencias negativas para la salud. Incluimos 6350 comentarios que fueron clasificados en 20 códigos agrupados en seis categorías: (1) violencia y discriminación, (2) salud y servicios de salud, (3) modo de elección del nacimiento, (4) incredulidad ante la información sobre la cesárea, (5) aborto y (6) descontento con el gobierno.

**Conclusiones:**

Los artículos incluidos no promovieron la cesárea sobre el parto vaginal, y los riesgos y consecuencias se representaron mayormente con precisión. Las percepciones sobre la representación de la cesárea mostraron un fuerte descontento y desconfianza hacia los médicos y el sistema de salud mexicano, así como incredulidad de los usuarios de Facebook hacia información factual sobre las consecuencias de la cesárea. Adicionalmente, este estudio documentó inequidad de género y violencia contra las mujeres, resaltando la necesidad de un enfoque de derechos humanos para el trato adecuado hacia las mujeres durante el embarazo y parto en México. Los resultados de este estudio contribuyen al campo emergente del análisis de redes sociales, y demuestran áreas donde se puede mejorar la comunicación en salud dentro de redes sociales.

## Background

Cesarean section is a life-saving intervention when vaginal birth represents a risk to the health of the woman or baby [1]. It is one of the life-saving services, or ‘signal functions’, that define a health facility regarding its capacity to treat obstetric and newborn emergencies [2]. Globally, rates of cesarean sections are increasing; in the last 15 years global rates of cesarean section have doubled to 21% of all births, and it is estimated that it will increase to 28.5% by 2030 [3, 4]. With population-level rates above 15%, there is no evidence that cesarean section is linked to an improvement of maternal or child health outcomes, and it can cause harm [5]. Although cesarean section is a life-saving intervention when medically indicated, it is not without risks for both women and babies, including higher risk for women of mortality, hemorrhage, infection, urinary and intestinal injuries, as well as long-term risks of abnormal placentation, ectopic pregnancy, stillbirth, and uterine rupture which increase after each subsequent cesarean section [6, 7]. Recent evidence also shows that the neonatal physiology of babies born by cesarean section can be altered by the difference in exposure to hormones and bacteria compared to those born vaginally with evidence suggesting a link with higher risk of allergies, atopies, asthma, and childhood obesity, and decreased gut microbiome diversity [6].

There are several reasons for increasing rates and include medical and non-medical factors. The so-called non-medical factors have been growing in importance during the last decades [5, 8]. Jenabi et al. conducted a systematic review to explore reasons for elective cesarean section on maternal request, and found the main reasons were fear of labor pain, anxiety for injury or death of the baby, negative experiences during prior births, anxiety for gynecology examination, desire to avoid long labor, anxiety for lack of support from the staff, and doctor suggestion, amongst others [9]. Other non-medical factors include convenience of scheduling a caesarean, provider’s fear of litigation, policies and financing, cultural norms, and perceptions or quality of health care in the country [5].

Women are exposed to multiple sources of information about pregnancy and birth that can influence their opinions and choices, including friends, families, and the media. These formal and informal sources can directly and indirectly influence their levels of fear, anxiety, and empowerment [10]. The quality and reliability of the information is crucial to elicit accurate views and expectations. In Spain and Brazil, research has explored the portrayal of cesarean section in women’s magazines and web-based resources [7, 11, 12]. In Brazil, Torloni et al. found that sources for the articles from women’s magazines were mostly journalists rather than health professionals or scientific experts, the overall portrayal was neither negative nor positive, but the portrayal of the risk of cesarean section might be misleading towards a lower-than-expected risk and particularly omitting longer-term risks [7]. The study from Spain produced similar results showing that the content of articles about cesarean section did not have comprehensive information and did not accurately represent the risks or benefits of cesarean sections, with only one-third of articles presenting objective medical or scientific information [11]. The research on web-based resources by Fioretti et al. in Brazil found that web pages in Portuguese regarding cesarean section had poor to moderate quality and completeness [12].

Between 2008 and 2017 in Mexico, on average 45.3% of all births were by cesarean section, making it one of the countries with the highest cesarean sections rates in the world [13]. Individual-level factors associated with cesarean section in Mexico include high education and socioeconomic status of women, early antenatal check-ups, and childbirth in private hospitals [14–16]. In addition, Indigenous women in Mexico are more likely to live in poorer municipalities with lower education levels, and are also experiencing increasingly high rates of cesarean section, coinciding with the introduction of universal health coverage and consequential increase of hospital births in those communities [1, 17, 18]. Recent research exploring discrimination and violence in maternity wards in Mexican public hospitals found that healthcare professionals held discriminative attitudes toward women, clients they perceived as ignorant, and people living in poverty [19]. Indigenous Mexican women are at heightened risk of discrimination and violation of their reproductive rights based on the intersection of oppressive social systems, class, gender and ethnic background [18]. There is growing evidence that relates unnecessary cesarean sections to obstetric violence in Mexico, due to power imbalances between women and health providers, caused by a strong tradition of sexism, and hierarchies in the medical environment [19–22]. Obstetric violence is a prominant structural issue in Mexico, and has recently received national legal recognition as a form of violence against women defined as “any action or omission from medical or administrative personnel belonging to public or private health services…that physically or psychologically harms, discriminates or denigrates women during pregnancy, childbirth and puerperium” [23]. Beyond harm and abuse, the overmedicalization of childbirth and disregard of women’s rights to bodily autonomy during maternity care can also be classified as a form of obstetric violence [24].

Outside of research studying healthcare services there is limited evidence considering the wider social context in which cesarean sections occur in Mexico, such as the influence of mass media on the portrayal of cesarean sections, which might affect the high rates by influencing the views and opinions of women, health service providers and society. Increasingly, people across the world are using social media to access news and information; for example, in the United States about half of adults report using social media as a news source, most often Facebook [25]. Facebook has become an ubiquitous social media platform, with almost 3 billion active global users in 2021, including over 80 million in Mexico where it is the most popular social media platform [26]. Against this background, our study aims to improve the understanding of the socio-cultural context of childbirth in Mexico by exploring the portrayal and perception of Cesarean section in Facebook media pages. Specifically, we aimed to (1) analyze the content of mass media articles shared on Facebook by top Mexican media outlets to understand how cesarean section is portrayed, and (2) analyze the perceptions about the portrayal of cesarean section using the comment threads attached to each Facebook post sharing the publication of the selected articles.

## Methods

This is a mixed-methods social media analysis using publicly available data from Facebook. We used a constructivist and interpretivist paradigm, which considers reality to be subjective and knowledge to be socially constructed by the meaning individuals or groups give to events or actions [27]. The constructivist and interpretivist paradigm focuses on understanding and interpretation of social realities, and is characterized by a naturalistic approach, meaning research data is collected from day-to-day settings [28]. The setting of this study is online Mexican social media, specifically Facebook which is used by approximately 61% of Mexicans [26]. Facebook is often part of the regular life of its users, and therefore has been recognized as a useful tool to observe behaviors and experiences in a naturalistic way, which is congruent with the constructivist and interpretivist paradigm [29]. For example, a 2019 survey found that 48% of Mexican Facebook users spent 2–3 h/day on the social media platform, and between 64 and 76% of Mexican news consumers use Facebook to read and interact with news [30, 31].

To meet the research objectives, we collected textual data, an unobtrusive method which extracts meaning from resources that already exist in the form of written documents [32]. The written documents collected were online publications from Mexican Facebook general news and other media pages and comment threads from the articles posts on Facebook. Therefore, data collection occurred in three steps (outlined below): (1) identification of media pages, (2) identification of relevant media articles, and (3) extraction of comment thread from each article publication on Facebook.

### Step 1: identifying media pages

We searched Facebook for Mexican media pages using the analytic online tool Socialbakers® (Astute, Inc, Prague, Czechia), filtering for Mexico as the country of registration, listed under the category “Media” and the subcategory “All Media”. The top ten pages with the largest audiences were selected based on the number of people who “liked” the page as of July 2019 (Table 1). Pages were included when most of their content was news, science, politics, parenting, or specific to a women’s or girls’ magazine. Pages were excluded when most of their content was sports, specific to either television or radio channel programming, specific to a product (such as the page for Facebook itself), comedy, music, cooking, as well as pages dedicated exclusively to celebrity gossip and entertainment.

**Table 1:** Top 10 Facebook pages, based on the number of likes

Position*	Facebook page	Audience**	Type of media
1	Muy Interesante México	8 233 792 likes	Science and nature magazine
2	Azteca Noticias	8 007 094 likes	News
3	Noticieros Televisa	6 849 077 likes	News
4	Revista Tú México	6 751 554 likes	Girls magazine
5	Revista Padres e Hijos (currently known as Vanidades Familia)	6 437 548 likes	Parenting magazine
6	Periódico El Debate (currently known as Debate)	5 392 132 likes	News
7	Sdpnoticias	5 053 001 likes	News
8	Revista Proceso	4 845 049 likes	News
9	El Universal Online	4 377 442 likes	News
10	Yahoo México	3 665 817 likes	News

*Position listed from the largest to smallest audience

**Audience as of July 5, 2019

### Step 2: identifying media articles

Media articles from the media pages selected in step 1 were identified using the function on Facebook to search for publications with the key Spanish word “cesárea” (cesarean section) and its common misspellings (sesarea, cesarea), selecting “Posts” as category, filtering the results by choosing as source each one of the ten selected media pages as “Post From”, all posts as “Post Type”, with anywhere as the “Tagged Location”, and January 2014 to June 2019 as “Date Posted”. The date range was selected due to the changing nature of the Facebook structure to obtain the most current and relevant data; for example, prior to 2014, media pages had a different format and were not used as widely. All text publications with a functional link to the full text article containing the word “cesárea” or its common misspellings, in either the publication header, the article’s headliner or the preview text from the article were included. Publications with content not relevant to cesarean section were excluded, as well as those containing only video content, and publications based on images only and not text, and publications with content regarding cesarean section in animals without relating it to humans.

### Step 3: extracting comments

We extracted all comment threads from each included media post, using the online tool ExportComments© (ExportComments, Romania) which automatically excludes comments that are only advertising or spam. Where there were multiple posts of the same article, we extracted comment threads from each post.

### Data management and analysis

All identified articles selected were saved as PDF for analysis and indexed by which Facebook page they were posted to and by date posted. A table was created summarizing content of all articles (Additional file 1). All exported comment threads were saved as Microsoft Excel documents for analysis. All articles were published in Spanish, and the analysis was also conducted in Spanish. Translation to English was done by MVC at the time of writing, and for the included quotes.

We conducted a three-staged mixed-methods analysis. First, to study the portrayal of cesarean section, we conducted a qualitative thematic analysis of the media articles, to explore, identify, analyze and report patterns in the data [33]. The following steps were adapted for the qualitative thematic analysis based on Braun and Clarke [33]: (1) becoming familiar with the data through repeated reading of the articles, (2) generating codes systematically in the data, and selecting significant data for each code, (3) looking for possible themes through the collation of codes, (4) collecting significant data for each theme, (5) developing a codebook, and (6) reviewing, defining, and naming themes and subthemes.

Second, to study how the portrayal of cesarean section is perceived, we used inductive quantitative content analysis of the individual comments and comment threads attached to each article posting. The purpose of analyzing the comments was to explore (1) the reactions of Facebook users to the information provided by the articles about cesarean section and (2) Facebook users attitudes and beliefs regarding the sociocultural context in which cesarean sections occur. Quantitative content analysis is a method systematically describing the meaning of data through the identification of categories which represent explicit or inferred communication [34]. We decided to perform content analysis because the number of comments any social media article will have depends on how much exposure it receives, which is determined by many factors including how many times it was shared and time of the day it was posted. Therefore, we focused on the content of the comments, rather than the number of comments received on a post. We used an inductive quantitative content analysis approach, because there is limited relevant knowledge on how Facebook users would respond to these types of articles in Mexico [34]. Using an inductive approach based on Ji Young and Eun-Hee [34], we conducted content analysis by: (1) open coding and developing of categories from a sample of the comments, (2) manually and systematically categorizing and recording all comments into the codes, and (3) counting the number of comments by each code.

Lastly, we connected the qualitative thematic analysis of the articles with the quantitative content analysis of the comments, by listing the total number of comments by each code under the subthemes of media articles created during thematic analysis.

## Results

**Fig. 1:**
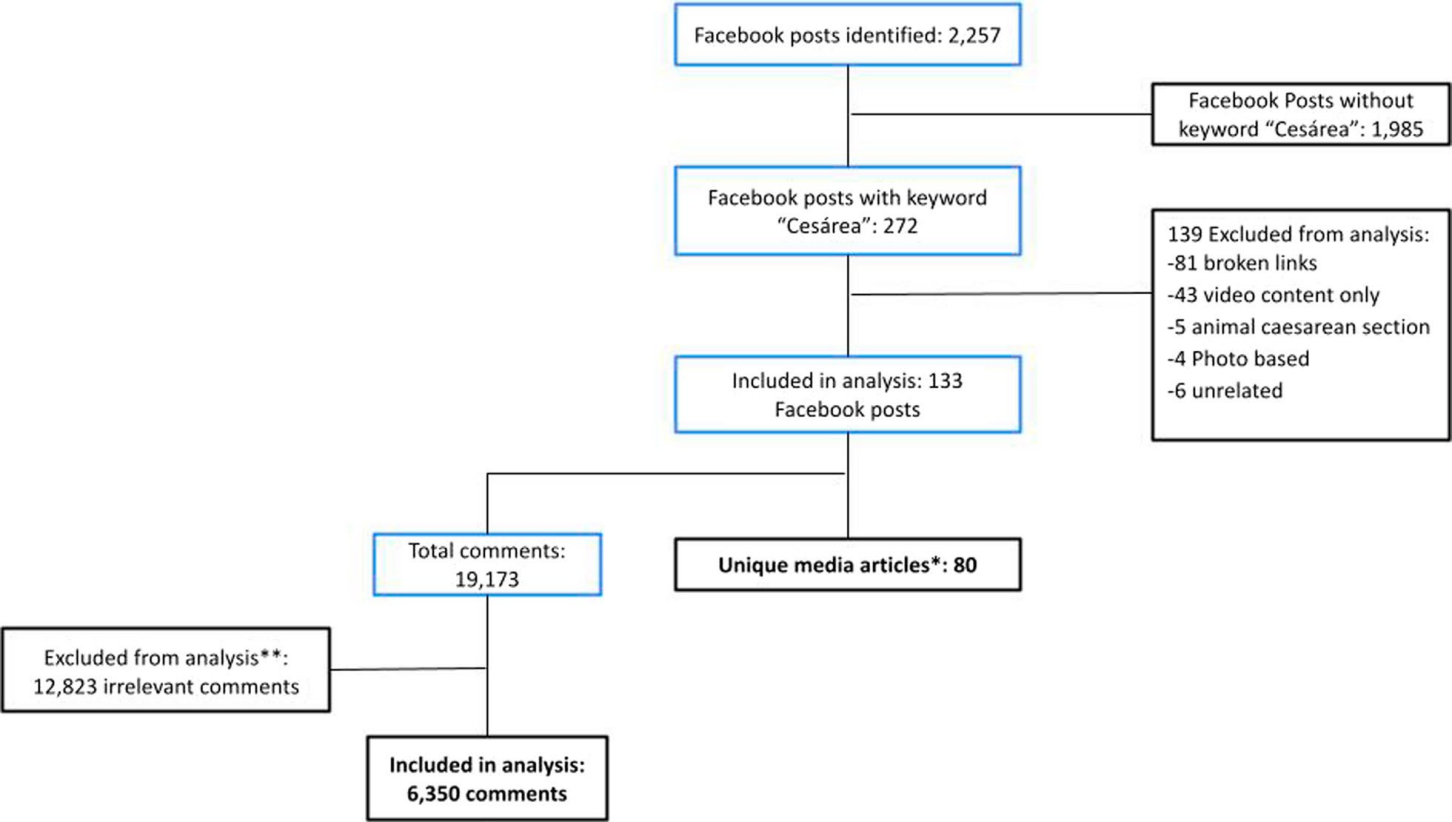
Search results of media articles and comments in selected Facebook pages. *Total of articles after merging duplicates. **Comments excluded when they did not provide any relevant information, were unrelated to the posts, or had ambiguous meaning (e.g., the name of a person, religious remarks only, jokes, laughter only, images only, duplicate comments, symbols or emojis only.)

Ten Facebook media pages were identified: seven general news, one science and nature magazine, one girls magazine and one parenting magazine. These media pages had audiences ranging from 3 to 8 million users each (Table 1). From these ten Facebook media pages, 2257 posts were identified between January 2014 and June 2019 (Fig. 1). 1985 posts were excluded for not containing “cesárea” in the heading, headliner, or preview text of the post. After merging duplicate articles (articles posted more than once on the same media page) and those that did not meet the inclusion criteria, 80 unique articles from 133 posts were included in the thematic analysis (Additional file 1: Summary table of included articles).

A total of 19,173 comments were extracted from 133 included posts, and 6350 comments were included in content analysis (median comments per article: 47, IQR 8–108). The 12,823 comments were excluded for not providing any relevant information, being unrelated to the post, or having ambiguous meaning. For example, this included comments with only: the name of a person ‘tagged’, religious remarks only (e.g., “amen”), jokes, laughter only (e.g., ‘ha-ha’), images only, duplicate comments, and symbols or emojis only, as evidence has shown people have variable interpretations of the meanings behind different emojis (Miller, 2018).

### Thematic analysis of included articles

Media articles reported on cesarean section in different countries, but predominantly in Mexico and Latin America. Some included articles only mentioned cesarean sections and were not specifically about the intervention per se; these articles were included because they document the socio-cultural context in which cesarean sections occur. We identified three major themes, depicted in Table 2: (1) Information about cesarean section, (2) Inequality and violence against women, and (3) Governance failure. Some articles contributed to more than one theme, for thematic analysis all themes and subthemes of each article were considered (Additional file 1).

**Table 2:** Key themes and sub-themes from the thematic analysis of articles

Theme	Sub-theme	Definition	Example of proper use
1. Information about cesarean sections	1.1 Vaginal birth as a better option than cesarean section	Any comparison between vaginal birth and cesarean sections in which the former is portrayed as a preferable option	“(vaginal birth) is less risky for the mom”
	1.2 Advice for recovery	Any advice regarding after care and recovery from cesarean sections	“Avoid sudden movements”
	1.3 Consequences for women	Any mentioned of risks and consequences of cesarean women affecting the health or wellbeing of women	“Risk of bleeding, infection and organ failure”
	1.4 Consequences for babies	Any mention of risks and consequences of cesarean section affecting the health or wellbeing of babies	“Increased risk of asthma, allergies and obesity”
	1.5 Cesarean section as a lifesaving procedure	Any case in which cesarean section saved the life of woman or baby	“Performed post-mortem cesarean section and managed to save baby”
2. Inequality and violence against women	2.1 Violation of reproductive rights	Any violation to the rights of women related to reproduction such as access to adequate reproduction health services, respect of bodily autonomy and consent for medical procedures or treatment regarding reproduction	“During the surgical procedure (cesarean section) they placed an Intrauterine device (IUD) without her consent”
	2.2 Discrimination	Any type of discrimination against women due amongst others to their ethnicity, Indigenous status, income, education, migration status, or gender	“Mexican immigrant handcuffed during childbirth”
	2.3 Objectification	Any reference where the female body is seen as an object	“Show your cesarean section scar and get free beer!”
	2.4 Women’s worth	Any reference to women’s worth being affected after having a cesarean section	“Pregnant women are less of a mother when they deliver through cesarean section”
	2.5 Body Image	Any reference to cesarean section or pregnancy affecting body image	“She doesn’t want a scar to stop her from looking perfect”
	2.6 Sexual violence	Any type of sexual abuse or harassment against women or girls	“36-year-old man likely responsible of statutory rape of her 13-year-old niece”
	2.7 Bodily harm	The physical harm or death of women due to deliberate violence against them	“25-year-old pregnant woman died in hospital…her brother sprayed her with gasoline and set her on fire”
3. Governance failure	3.1 Medical negligence	Any medical malpractice during cesarean section resulting in injury or harm to woman or baby	“Woman visited hospital for a cesarean section, suffered from second degree burns in her legs after surgery”
	3.2 Impunity	Any stance where people or institutions responsible for human rights violations are not held accountable by the justice system	“Anesthesiologist found responsible of not watching oxygen levels of woman during cesarean section contested the charges and walked free”
	3.3 Unnecessary cesarean section	Any mention of cesarean sections being unnecessary or exceeding the appropriate rates	“Half of Mexicans are born through cesarean sections”

1. **Information about cesarean section**
  This theme explores the portrayal of cesarean section as information communicated by the Mexican media regarding this procedure, such as any article that was informative or educational regarding cesarean sections. A total of 45 articles contributed to this theme (Additional file 1) and five sub-themes were identified: (1) vaginal birth as a better option than cesarean section, (2) advice for recovery, (3) consequences for women, (4) consequences for babies, and (5) cesarean section as a lifesaving procedure.
2. Vaginal birth as a better option than cesarean section
  *“It’s the most organic way*” (article 52)
  When compared with cesarean section, vaginal birth was generally reported as a less risky option for the woman, and beneficial for the baby. For women, vaginal birth was portrayed as having a lower risk of mortality and infection, facilitating breastfeeding, and having a faster recovery than cesarean section (articles 15 and 58). Traditional midwives (non-professional community health workers who follow Mexican Indigenous medicine) also referred to vaginal birth as a better option for women and blamed hospitals for the high rates of cesarean sections (article 13). Several articles also emphasized the advantages of vaginal birth in reducing the risk of asthma, allergies, and obesity, stating that the exposure of specific bacteria and hormones during labor and childbirth was a protective factor (articles 5, 18, 53, 58). Another reason given for vaginal birth as a better option was the claim it allowed for ‘natural selection’ of the healthiest women and babies surviving childbirth, implying that women with narrow hips or babies too large were not the most appropriate for the survival of the human species and cesarean sections were changing evolution (articles 17 and 79). These results present vaginal birth as the most desirable mode of birth, and cesarean sections as a procedure that should be used only in emergencies.
3. Advice for recovery
  *“Keep the wound clean and dry”* (article 59)
  The articles in this subtheme communicated clearly that cesarean section was a surgical procedure that was painful and required special care for prolonged time in comparison to vaginal birth. The information provided was accurate and useful, most of the articles providing advice were published in the parenting magazine (Revista Padres e Hijos). Most advice for recovery from cesarean section was focused on preventing infection, reducing post-operative pain, and avoiding constipation. The most common advice was keeping proper hygiene using soap and water to clean the wound (articles 9, 44, 55 and 59), and using a light girdle or body shaper once the wound was healed but only if women wanted it to feel more comfortable (articles 55, 59 and 60). The articles promoting the use of a body shaper emphasized that it would reduce abdominal muscle tone if overused (articles 55, 59 and 60). To help cope with post-operative pain, the following options were recommended: breastfeeding (article 45), analgesics (articles 55 and 61), avoiding heavy lifting and sudden movements, finding a comfortable position when breastfeeding (articles 44 and 59), and applying pressure to the wound when laughing or coughing (article 55). If nausea or vomiting occurred, it was advised to consult with a medical professional immediately (article 62). To avoid constipation, it was advised to avoid astringent foods (e.g., rice and potato) and increase water and fiber intake (article 9). Articles also reminded women that recovery from cesarean section took longer than vaginal birth (articles 60 and 62). One article mentioned that most private hospitals would encourage cesarean sections as they could charge more for the procedure and advised pregnant women to take this into consideration (article 60).
4. Consequences for women
  *“Because the incision is in the abdomen, laughing, coughing, sneezing or even leaving bed will hurt”* (article 15)
  The consequences of cesarean sections for women highlighted in the articles were higher risk of death, placenta previa, placenta accreta, infection, bleeding, pulmonary embolism, blood clotting (articles 10, 15 and 57), increased risk of miscarriage or stillbirth in subsequent pregnancies (articles 28, 43 and 57), pain (article 15 and 79), constipation (article 62), post-traumatic stress disorder (article 56), rupture of stitches (article 38) uterine scarring, pelvic prolapse, and urinary incontinence (articles 28 and 57). The portrayal of the consequences of cesarean section for women were congruent with currently accepted current medical evidence.
5. Consequences for babies
  Included articles mentioned the potential consequences of cesarean sections for babies, including higher risk of obesity, asthma, and allergies, compared to babies born by vaginal birth (articles 5, 15, 17, 28, 43 and 76). However, not all claims of the consequences of cesarean sections in babies were accurate. For example, one article reported on a study linking neurological alterations in mice born by cesarean section suggested the same neurological alterations happened in humans and lead to autism, without referencing the specific study described or having supporting evidence for this effect in human populations (article 39). Other articles reporting on scientific studies of mice born by cesarean or vaginal birth suggested that child brain development was affected by cesarean section, because of differences found in brain activity immediately prior to birth (articles 18 and 53) but did not provide reference to the specific studies being described for this effect in humans.
6. Cesarean section as a lifesaving procedure
  This subtheme was commonly present in articles from the previous subthemes. One-quarter of all included articles described cesarean section as a lifesaving procedure for the woman and baby in emergency circumstances due to complications during pregnancy or childbirth or in cases were the woman died but cesarean sections saved the baby (article 1, 4, 14 and 25). In two articles from Portugal and Mexico, caesarean sections were performed on women who were kept on life support until babies had developed enough to survive outside of the uterus (articles 41 and 42).
7. **Inequality and violence against wome**n
  Cesarean section was commonly mentioned when reporting on cases of social and gender inequality as well as violence against women, 33 articles contributed to this theme (Additional file 1) and 7 subthemes were identified: (1) violation of reproductive rights, (2) discrimination, (3) objectification of women, (4) women’s worth related to cesarean section, (5) body image, (6) sexual violence, and (7) bodily harm of women.
8. Violation of reproductive rights
  *“I told the doctor I wanted a “natural birth” and she laughed at me, she said I had no idea what a first childbirth was, that it would hurt a lot, that I could tear, and even that my bladder would fall” *(article 10)
  Different types of violations of reproductive rights related to cesarean section were found. There were articles reporting on long term contraception implementation in Mexican Indigenous women in Mexico without informed consent during cesarean sections (articles 2, 67). Another article reported on pregnant women being coerced by doctors to have cesarean sections in Mexico (article 10). Several articles reported on the denial of abortion and forced pregnancy of adolescent girls in Argentina (articles 11, 12, 19, 27, 34 and 65) and Paraguay (articles 16, 50). One article reported a case in England of a person who was intersex who had their genitals removed non-consensually during a cesarean section (article 75).
9. Discrimination
  *“They (in the public health system) treat you like an ill woman, that knows nothing about her body, and it seems like you are not allowed to ask…”* (article 10)
  There were reports of Mexican women being discriminated and abused during cesarean sections due to their Indigenous status, immigration status, and their gender. Two articles reported on the case of an Indigenous woman and her family who received inadequate care during and after cesarean section (article 2 and 67). An article reported on a Mexican woman handcuffed during a cesarean section in the United States of America (article 49). Several articles reported on women being undermined and discriminated against based on their gender in Mexico (article 10, 36, and 66).
10. Objectification
  Objectification of women’s bodies was present in two articles reporting on a bar offering free beer to women if they showed their cesarean section in a Mother’s Day celebration in Mexico (article 64 and 70). The coverage of this promotion emphasized the sexism of asking women to show their bodies for a material gain, as well as reporting on feminist groups denouncing the gender violence of objectifying women’s bodies.
11. Women’s worth
  There were two main cases reported on the media in Mexico where women’s worth was implied to be diminished after having a cesarean section. The governor of a state in northern Mexico who was aiming to promote vaginal births made a statement saying that a woman who avoids cesarean section is more of a mother (article 36), which led to a congresswoman reporting the governor to the National Human Rights Commission for sexism (article 66). The other case involved a woman who was kidnapped outside a metro station in Mexico City (most likely for sexual trafficking) and released when the captors noted her cesarean section scar claiming she was “useless” now (articles 8 and 34).
12. Body image
  A few articles implied body image issues related to cesarean section and pregnancy. The scar of cesarean section was reported as something to hide (article 8), or as an unattractive feature (article 68). There were also mentions of an international celebrity who pressured herself to lose weight after her first cesarean section (article 46, 63).
13. Sexual violence
  Sexual violence resulting in pregnancy and subsequently in cesarean section was reported to have occurred in Mexico (article 51), Paraguay (articles 16 and 50) and Argentina (article 11, 12, 19, 20, 33, 34, 35, 74). All previously mentioned cases had an adult relative as the perpetrator of the crime, and the people who experienced the abuse were all young teenage girls.
  The senate debate about the legalization of abortion in Argentina in 2018 and consequential social disruptions were widely covered in the Mexican media. Two young girls aged 11 and 12 who were eligible to have legal termination of pregnancy had the procedure delayed to a point where it was no longer legal, forcing the young girls to have a cesarean section to premature babies in what activist groups denounced as “torture” (article 12). These cases, amongst similar ones, were made emblematic and were heavily reported about in the media as protests and massive marches occurred in Argentina fighting for the right to choose.
14. Bodily harm
  There were three cases of bodily harm of pregnant women that resulted in emergency cesarean section. In Mexico, a woman was purposefully burnt with gasoline by her brother (article 14), and another woman was shot due to drug cartel violence (article 25). In Brazil, a woman was shot due to a lost bullet (article 1). The articles focused on the survival of the babies and barely discussed the women or caesarean section.
15. **Governance failure**
  This theme includes articles where cesarean sections were related to ineffectiveness of government institutions such as the public health and justice system, 27 articles contributed to this theme (Additional file 1), and 3 subthemes where identified: (1) medical negligence, (2) impunity, and (3) unnecessary cesarean sections.
16. Medical negligence
  Medical negligence was a common subtheme in the included articles. All 9 reported cases of medical negligence related to cesarean sections in Mexico occurred in places with large rural and Indigenous populations. The consequences of the medical negligence varied: An Indigenous woman has remained in a vegetative state after surgery for more than a decade (articles 2 and 66), a baby died after woman was denied a cesarean section (article 48). The death of an Indigenous woman 5 days after the surgery (article 6), the death of both woman and baby after unsupervised medical residents and interns performed a cesarean section (article 24). A woman had her legs accidentally burn during surgery (article 21), and the neurological damage to a baby (article 3).
  An article stated that according to the National Institute of Geography and Statistics (INEGI), about 65% of fetal deaths in Mexico in 2016 were due to negligence by an obstetric doctor, and the remaining 35% due to negligence by other doctors, nurses, or midwives (article 13). However, this article incorrectly reported information from INEGI, and these numbers actually represent the total number of fetal deaths based on the type of health provider attending the birth—not due to negligence.
  There were also reports of international negligence cases. A doctor in the United Kingdom refused to perform a cesarean section forcing vaginal birth manually and accidentally decapitating the baby (article 40, 69 and 75). In India, a doctor who was intoxicated with alcohol performed a cesarean section resulting in death of both woman and baby (article 23). In Spain, a woman died after cesarean section and the baby was born with a scalpel injury (article 22). An elective cesarean section In Argentina led to the death of a healthy young woman (article 37). In Panama, scissors were left in a woman’s uterus after a cesarean section (article 73). Similarly, in Jordan a cell phone was allegedly left behind inside a woman’s abdomen after the surgery (article 72).
17. Impunity
  Impunity is a major issue in Mexican society. There were articles reporting impunity cases after medical negligence during or after cesarean section: The family and fellow community members of an Indigenous woman who died after a cesarean section peacefully protested to demand an investigation into her death (article 6), no restorative justice was given to the family of an Indigenous woman who has been in a vegetative state for over 10 years (article 2), and nobody was held responsible for the burns of a woman during a cesarean section nor follow up treatment was provided for her injuries (article 21).
18. Unnecessary cesarean sections
  *“Latin America reigns in a world plagued by unnecessary cesarean sections”* (article 77)
  Several articles reported on the high rates of cesarean sections and clearly stated the procedure should only be used in emergency situations when the life of woman or baby are at risk. Three articles mentioned the high rates of unnecessary cesarean sections to be alarming or referred to it as a worldwide epidemy (articles 29, 54 and 77). An article claimed the reasons for high cesarean sections rates in Latina America to be women trying to avoid pain, doctors aiming to schedule or reduce the duration of childbirths, as well as lack of fiscal inspections (article 77). One article that included interviews of medical providers mentioned that private hospitals were promoting unnecessary cesarean sections with their clients and rarely performed vaginal births, but provided no evidence for this claim (article 57). Another article reported half of Mexicans are born through cesarean section and stated the excess of this procedure was related to not having enough beds for women going through lengthy vaginal births in public hospitals, and private hospitals aiming to charge higher fees by promoting cesarean sections over vaginal births (article 10).

### Content analysis of comments from media posts

**Fig. 2:**
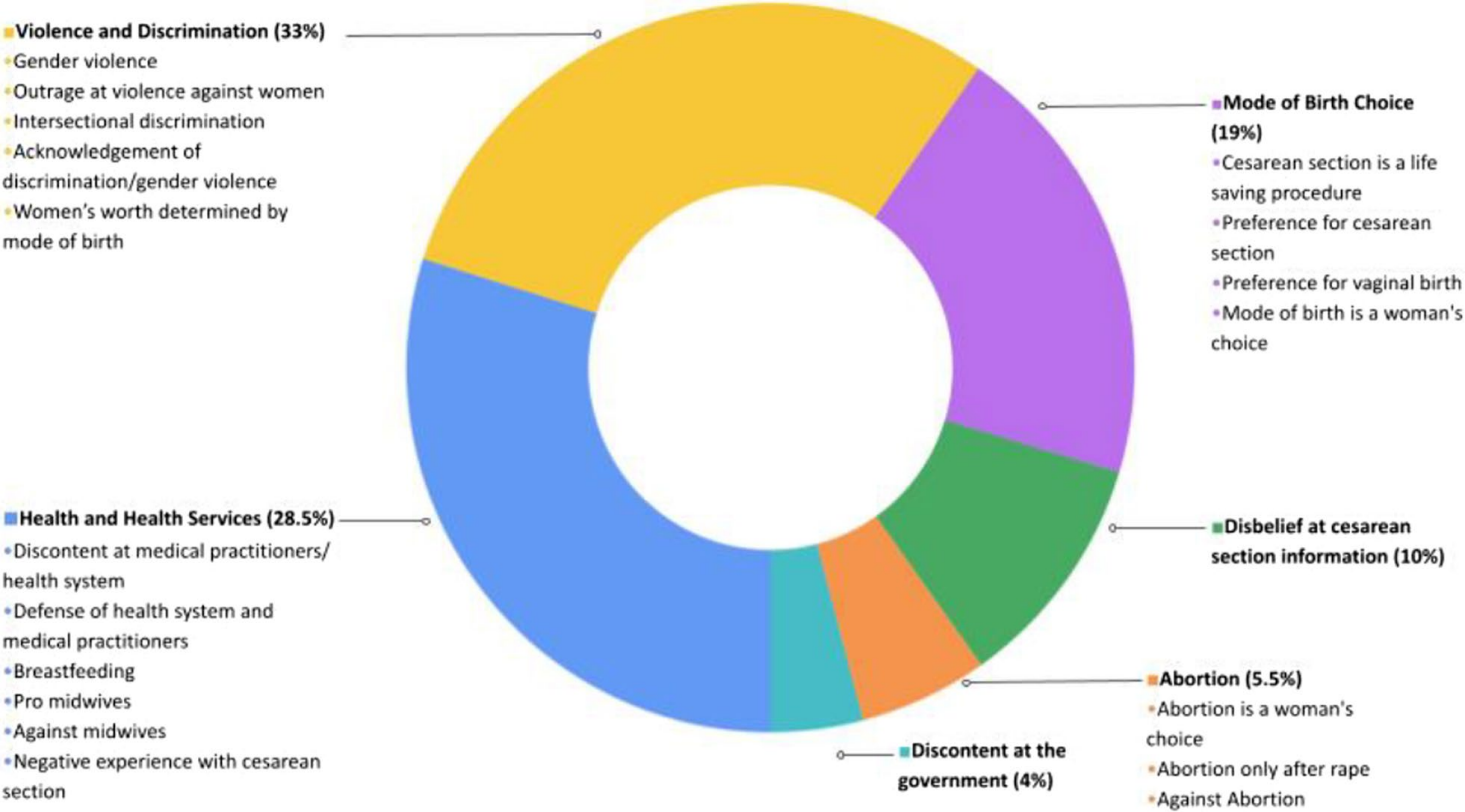
Code domain distribution of comments from Facebook media posts

Comments from all included articles were analyzed. All included comments (n = 6350) were classified into one of 20 major categories corresponding to the content of most of the text of the comment. Table 3 describes the code groupings, frequency of use, and examples of comments classified within the code. The content of included comments from media posts was distributed across 6 main code domains (Fig. 2). The largest domains of comments were about “violence and discrimination” (33.0% of comments, grouping 5 codes), “health and health services “(28.5% of comments, grouping 6 codes) and “mode of birth choice” (19.0% comments, grouping 4 codes).

**Table 3:** Comment codes from included Facebook media posts, examples of use and number of comments by code

Major category	Code name	Examples of proper use	n (%) comments (n = 6350)
Violence and discrimination	Outrage at violence against women	“This is horrible, what has society come to?”	997 (15.7%)
	Gender violence	“Those horny girls only bring children to the world to suffer” “…Contraception after birth should be mandatory, there is women that after a year are having another child and that is when complications arise”	665 (10.5%)
	Acknowledgement of discrimination or gender violence	“They are discriminating the patient because she is Indigenous, where are human rights?” “Violence against women has been so normalized that now when women come forward to report it, they experience double victimization”	241 (3.8%)
	Intersectional discrimination	“If they cannot afford children, why do they get pregnant?” “Women in those communities never go to prenatal care or look after their reproductive health, having babies like rabbits and of course those pregnancies have complications and because they are ignorant, they don’t take responsibility”	106 (1.7%)
	Women’s worth determined by mode of birth	“a real woman gives birth (vaginally) and doesn’t whine about it” “…I’m grateful my wife had a cesarean section since a part of her remains perfect and without damage…” “Women are not as brave as they used to be…”	103 (1.6%)
Health and health services	Discontent at medical practitioners/health system	“It is known that doctors do unnecessary cesarean sections so they can charge more for it” “So many horror stories from the public health system” “In the public health system, they help you…die sooner”	1366 (21.5%)
	Defense of health system and medical practitioners	“Doctors are human not gods, all surgeries have risks” “…Clinical staff barely rest, there is not enough hospitals for the population, which makes timely care difficult”	175 (2.8%)
	Negative experience with caesarean section	“Because of a poor performed cesarean section I had to have my uterus removed” “I had a cesarean and suffered a lot, they performed it on me because my mother wasn’t with me, I was alone”	159 (2.5%)
	Breastfeeding	“…I cry whenever I think I can’t breastfeed because of having a necessary cesarean section, it wasn’t my choice” “I’m happy to donate my breastmilk to the baby that needs it”	54 (0.9%)
	Pro midwives	“A good (traditional) midwife is the best way; women don’t die on their watch because they can detect complications and refer them to a doctor if needed”	24 (0.4%)
	Against midwives	“Preferring a (traditional) midwife instead of a hospital birth is like preferring to get smallpox instead of a vaccine”	24 (0.4%)
Mode of birth choice	Cesarean section is a lifesaving procedure	“Cesarean sections are not by choice; they exist to save a baby’s life when it’s at risk…”	445 (7.0%)
	Preference for vaginal birth	“I have had both a CS and natural birth, I prefer natural birth by a thousand” “I had a natural birth and I recovered quicker”	275 (4.3%)
	Preference for Cesarean section	“I love cesarean sections, the CS is my friend, I’m glad I’m alive now and not in the past” “I chose a CS, it’s a beautiful experience, I don’t regret it and I recovered in less than 3 weeks!”	257 (4.0%)
	Mode of birth is a woman’s choice	“Every woman is free to choose (their mode of birth)”	229 (3.6%)
Disbelief at caesarean section information	Disbelief at Information about Cesarean section	“This is a lie; my children were born through cesarean section and they are very smart”	636 (10.0%)
Abortion	Abortion only after rape	“I’m against abortion but in these cases (sexual violence) I support it should be legal” “I believe abortion should be legal when there is rape, not when (pregnancy) is out of horniness”	123 (1.9%)
	Abortion is a woman’s choice	“Yes, to legal abortion” “Give women the right to choose if they don’t want to be mothers”	118 (1.9%)
	Against abortion	“There is nothing to debate, abortion is murder” “Say no to abortion, yes to life”	105 (1.7%)
Discontent at government	Discontent at the government	“The public health system could compete with the private sector if it weren’t because the government steals the funds” “Justice doesn’t exist in Mexico”	248 (3.9%)

The most frequent code across all comments was “discontent at medical practitioners/health system” (n = 1366, 21.5%), where comments centered around perceived poor quality care at public hospitals and poor healthcare provider behavior. The second most frequent code was “outrage about violence against women” (n = 997, 15.7%), where users responded in support of women and with indignation to incidences of violence. This contrasted with the third most frequent code (“gender violence” (n = 665, 10.5%), where user comments perpetrated violent and misogynistic threats in response to article content or to other users. User comments were also commonly related to “disbelief at information about cesarean section” (n = 636, 10.0%), where comments centered around dismissal of health information, typically due to differences with their own experiences or the experiences of people within their social networks. In contrast, “cesarean section is a lifesaving procedure" (n = 445, 7.0%), was also common for users to reiterate the importance of maternity care services including cesarean section.

### Content analysis of comments by media article subthemes

Since most articles were classified into more than one subtheme, we used the main subtheme classification of each article (Additional file 1) to then map the content analysis of the comments, in order to explore the relationship between the content of the article and the type of responses in the comments (Additional file 2 shows the frequency of all comment codes by article subtheme). Table 4 shows a summary of the most frequent comment codes, by main article subtheme, in order to depict the most common types of interactions of Facebook users with the content of the articles. Overall, we found interesting patterns in the way that Facebook users interacted with the content. For example, articles classified as “consequences of caesarean section for women” were most likely to have comments related to “disbelief at information about cesarean section” (n = 212, 32.9%), and articles classified as “cesarean section as a lifesaving procedure were most likely to have comments related to “gender violence” (n = 149, 36.3%).

**Table 4:** Summary of most frequent content in comments by media article subthemes

Article theme	Article sub-theme	Total # comments in article sub-theme (n = 6350)	1st most frequent content code in comments	2nd most frequent content code in comments	3rd most frequent content code in comments
1. Information about cesarean sections	1.1 Vaginal birth as a better option than Cesarean Section	66	Against midwives (n = 20, 30.3%)	Pro midwives (n = 18, 27.3%)	Discontent at medical practitioners/health system (n = 8, 12%)
	1.2 Advice for recovery	34	Preference for vaginal birth (n = 10, 29.4%)	Preference for cesarean section (n = 9, 26.5%)	Breastfeeding (n = 5, 14.7%)
	1.3 Consequences for women	644	Disbelief at Information about Cesarean section (n = 212, 32.9%)	Cesarean section is a lifesaving procedure (n = 97, 15.1%)	Preference for Cesarean section (n = 93, 14.4%)
	1.4 Consequences for babies	611	Disbelief at Information about Cesarean section (n = 385, 63.0%)	Cesarean section is a lifesaving procedure (n = 78, 12.8%)	Preference for vaginal birth (n = 46, 7.5%)
	1.5 Cesarean section as a lifesaving procedure	410	Gender violence (n = 149, 36.3%)	Outrage at violence against women (n = 84, 20.5%)	Breastfeeding (n = 30, 7.3%)
2. Inequality and violence against women	2.1 Violation of reproductive rights	697	Outrage at violence against women (n = 243, 34.9%)	Abortion only after rape (n = 96, 13.6%)	Abortion is a woman’s choice (n = 87, 12.5%)
	2.2 Discrimination	0	No comments from Facebook media posts sharing articles with this main subtheme	n/a	n/a
	2.3 Objectification	194	Gender violence (n = 118, 60.8%)	Outrage at violence against women (n = 29, 14.9%)	Acknowledgement of discrimination or gender violence (n = 22, 11.3%)
	2.4 Women’s worth	702	Outrage at violence against women (n = 260, 37.0%)	Gender violence (n = 241, 34.3%)	Discontent at the government (n = 98, 14%)
	2.5 Body image	0	No comments from Facebook media posts sharing articles with this main subtheme	n/a	n/a
	2.6 Sexual violence	241	Outrage at violence against women (n = 62, 25.7%)	Acknowledgement of discrimination or gender violence (n = 38, 15.8%)	Gender violence (n = 33, 13.7%)
	2.7 Bodily harm	86	Outrage at violence against women (n = 75, 87.2%)	Gender violence (n = 6, 7.0%)	Discontent at the government (n = 5, 5.8%)
3. Governance failure	3.1 Medical negligence	1693	Discontent at medical practitioners/health system (n = 1040, 61.4%)	Outrage at violence against women (n = 236, 13.9%)	Defense of health system and medical practitioners (n = 153, 9.0%)
	3.2 Impunity	0	No Facebook media posts sharing articles with this main subtheme	n/a	n/a
	3.3 Unnecessary cesarean section	972	Discontent at medical practitioners/health system (n = 229, 23.6%)	Cesarean section is a lifesaving procedure (n = 202, 20.8%)	Mode of birth is a woman’s choice (n = 151, 15.5%)

Interesting patters also emerged in assessing articles related to inequality and violence against women, where comments coded as “outrage at violence against women” were most common on articles classified as “violation of reproductive rights” (n = 243, 34.9%), “women’s worth” (n = 260, 37.0%), “sexual violence” (n = 62,25.7%), and “bodily harm” (n = 75, 87.2%). In contrast, articles coded as “objectification” primarily had comments related to “gender violence” (n = 118, 60.8%).

Within the articles in the “governance failure” overarching theme, comments coded as “discontent at medical practitioners and the health system” were most common for both “medical negligence” (n = 1040, 61.4%) and “unnecessary cesarean section” (n = 229, 23.60%).

## Discussion

### The portrayal of cesarean section

Overall, articles shared on Facebook media pages did not appear to promote the procedure. The risks and consequences of cesarean section were mostly represented accurately, and it was only seen as a better option than vaginal birth when it was a medical emergency. However, there were a few articles that reported on academic studies inaccurately where large assumptions were made using misleading headlines, such as cesarean section causing autism or interfering with evolution. This type of misinformation can have profound impacts: in the case of the consequences of cesarean section for babies it may lead to pregnant women rejecting cesarean sections even when the procedure is medically necessary.

These results are different to what has been found in similar studies that explored portrayal of cesarean section in traditional media outlets and web-based sources. Torloni et al. [11] found in a 21-year review of Spanish women’s magazine that the portrayal of cesarean section provided insufficient information for the readers to fully understand the benefits and risks of the procedure. In a 20-year review of Brazilian women’s magazine, Torloni et al. [7] found the portrayal of cesarean sections did not favor vaginal birth nor cesarean section, unlike the portrayal in Mexican Facebook media pages, where vaginal birth was favored. It is important to note that this study explored a more recent portrayal and had an inductive approach to analysis. This contrasts with the other studies, which used pre-specified outcome measures, such as accuracy and comprehensive of information, which may explain some of the differences. Moreover, the authors of the media articles analyzed in this study were mostly anonymous, and only a few articles had interviews with experts or medical professionals. This is similar to what was found in the Brazilian research where most of the sources in the articles from women’s magazines were not optimal and the information on web-based sources about cesarean section had poor to moderate quality and completeness [7, 12].

Because of the inductive approach, our study identified that cesarean sections in Mexico were found to occur in a context of inequality, gross human rights violations against women and girls, as well as government failure. Reports about the procedure in Indigenous women often displayed a double discrimination in the Mexican health system due to their gender and Indigenous status. Rural and Indigenous women were commonly reported to experience medical negligence, which was linked to impunity. Gender inequality was also present in the articles where women’s worth was related to cesarean section, such as the statement by a Mexican governor saying women who have had cesarean section are less worthy mothers. A few articles that reported emergency cesarean section did not report on the well-being of women and prioritized babies; this may be interpreted as a portrayal where women are seen only as reproductive vessels whose value was determined by their birthing outcomes.

In all the media studied, vaginal birth was referred to as “parto natural” (natural birth) or “parto normal” (normal birth), common terms used in Mexico that inherently implies cesarean sections are “unnatural” or “abnormal”. The terms “natural” and “normal” may shame or stigmatize women who have had cesarean section. Some of the articles providing advice for the recovery after a cesarean section focused on scarring, which was portrayed as something unwanted, shameful, or to be hidden.

### The perceptions of cesarean section portrayal

We analyzed Facebook users’ comments on media posts about cesarean section to better understand how users reacted to and interacted with these posts, and reflect on the attitudes and beliefs that inform the sociocultural context in which cesarean sections occur. We found that social media users mostly rejected information about the risks of cesarean section for both women and babies based on their personal experiences, for example refusing to believe cesarean section may be linked to obesity in children because they consider their own children born through cesarean section to have a healthy weight.

Social media users in the comments discussing mode of birth often defended their choice for cesarean section by claiming cesarean section is less traumatic and safer for the baby compared to vaginal birth. To what extent this is a misconception and information campaigns would have the potential to fix it or it is the result of motivated reasoning bias (and information interventions would be insufficient) needs to be assessed. Motivated reasoning is a cognitive bias showing the tendency to believe and justify more readily what is more desirable to us, in this case, the avoidance of vaginal birth, for example, for fear of mistreatment during childbirth [35].

In comments addressing women’s worth related to mode of birth the content showed that social media users commonly considered women who choose a cesarean section as lazy, cowardly, or vain, whereas women who had vaginal births were stronger, “real women” or braver. The global concern for the increasing rate of caesarean section has fostered the interest to explore interventions to reduce overuse. While mass media campaigns are recommended to change population health behaviors, campaigns to reduce unnecessary caesarean section have not used key principles recommended for the creation and implementation of health communication interventions [36, 37]. The promotion of vaginal birth should not stigmatize women who have to undergo a cesarean section. We need to reconcile the understanding that vaginal birth is the best option in the absence of complications with the respect for women who need a cesarean section. Women’s worth does not lie in her ability or capacity to give birth vaginally and this message needs to be emphasized, and related behaviors should be built into Mexican society.

The content in the comments of Facebook posts suggest a strong discontent and distrust towards medical practitioners and the Mexican health system related to medical negligence and high rates of unnecessary cesarean section. We could not find any evidence in the scientific literature on the prevalence and incidence of medical negligence in maternal and child health in Mexico. However we did find one article showing that 15.6% of complains submitted to the National Commission of Medial Arbitration from 1998 to 2000 in Mexico involved obstetrics and gynecology, 57.6% of all complains were submitted by women, and 36.5% of complains were a result of malpractice in which lack of skill was the main reason (67.4%) [38]. This suggests that the distrust towards medical professionals in Mexico is not unfounded. As documented by Castro et al., the distrust on the health system results in some women delaying or avoiding medical care during pregnancy for fear of doctors or previous negative experiences with the health system, which can lead to maternal mortality [39].

Additionally, many comments expressed outrage about violence against women but only a few acknowledged this violence as a gender problem or discrimination. Moreover, gender violent content in comments was the third most common type of content compromising 10.5% of all comments, which is alarming but not surprising given the high rates of gendered violence in Mexican society [40, 41]. Comments frequently contained intersectional discrimination against people with low incomes and women were dehumanized and blamed for high fertility rates, poor health, or lower education levels. These comments are similar to the discriminatory views and attitudes of health care providers from public hospitals in Mexico documented by Santiago et al., suggesting widespread classism [19].

While cesarean section is a medical procedure, its use is also influenced by health system structures and social determinants of health, including individual and societal views of gender and women’s reproductive health and rights. Facebook users’ perspectives on the portrayal of cesarean section showed violence, discrimination, and trust of personal experience over evidence-based information. Our analysis contributes to better understanding of these social factors, and suggests that maternal health promotion and education may benefit from social media-based communication and sociobehavioral science strategies to influence attitudes towards women’s reproductive health.

## Strengths and limitations

This study has major strengths. We minimized bias by identifying major media outlets with millions of people in their audience and used rigorous and systematic methods to identify and screen relevant articles. We were also able to obtain the perspective and opinions of social media users through their public comments, something that would be difficult to obtain using more traditional data sources. Moreover, to our knowledge, this is the first study that has used social media research methods in this way, demonstrating how innovative social media analysis can yield fruitful insights into social aspects of health. Another strength is that the study of Facebook provides a very up-to-date window into the views of society compared with reviews of the scientific literature, for example, where publication times have a considerable time lag. This study main limitations are that it only studies one type of media: online textual media articles and comments, image-based media could have a strong persuasive power and was not included in this analysis. Additionally, it is unknown how much media influences decision making in Mexico regarding childbirth, and more research is needed to understand this relationship. Another limitation is that we found that Facebook posts reporting on other countries had comments showing that many media users thought the media articles were reporting on Mexico, which suggests that social media users did not read the full text of the media shared and comments may be based on the article preview or title rather than content. Moreover, we were unable to determine whether Facebook users were from or living in Mexico; however, we expect that most would be given that we selected the top media pages in Mexico and comments that clearly stated the user was from, and living in, another country were excluded.

## Implications for future research

There is a need for evidence about the main sources of information about pregnancy and delivery for people in Mexico, the impact of social media on views and attitudes about mode of birth, and evidence-based strategies to improve the delivery of information. Social media could be a useful outlet to address misinformation about childbirth and provide public health messaging to reduce unnecessary cesarean sections. There is also a need for research to understand women’s preferences and views of mode of birth in Mexico, and the extent to which those preferences are addressed by health providers. Additionally, considering the high level of distrust towards the health system in Mexico showed in our results, research is needed to assess the impact of negligence and suboptimal health systems structure and resources on maternal and perinatal health outcomes. Since evidence suggest that media campaigns to reduce caesarean sections are successful only when there is safe and supportive environments in place that can facilitate vaginal birth [36].

## Conclusions

In conclusion, there is accurate and useful information on social media outlets regarding cesarean section risks and consequences in Mexico, this could mean the high rates of this procedure might be more related to structural and behavioral issues in the public and private health system than to misinformation, as well as cultural standards that perpetuate gender discrimination. Considering the Mexican social context documented in this study, pregnancy and cesarean sections are events in which women are more likely to experience violence and gender inequality, therefore a human rights approach is critical for the appropriate treatment of women during childbirth. This is particularly true regarding Indigenous women who experience heightened discrimination and abuse. The findings from this study can also contribute to the emerging field of social media analysis, and demonstrates clear areas where social media science communication can be improved.

## Supplementary Information

**Additional file 1: **Summary table of included articles by subtheme, media page, year and story origin.

**Additional file 2: **Frequency of all comment codes in comments by article subtheme.

## Data Availability

The datasets used and/or analyzed during the current study are available from the corresponding author on reasonable request.

## Abbreviation

CS: Cesarean section
