# Supplementary material for: The portrayal and perceptions of cesarean section in Mexican media Facebook pages: a mixed-methods study
Source: Reprod Health. 2022 Feb 22;19:49. doi: 10.1186/s12978-022-01351-8 (PMC8862237; doi:10.1186/s12978-022-01351-8)
Supplement: Supplementary file 1 — Additional file 1: Summary table of included articles by subtheme, media page, year and story origin. [file 12978_2022_1351_MOESM1_ESM.docx]

Appendix S1. Summary table of included articles by subtheme, media page, year and story origin.

| **Article ID** | **Article title** | **Year** | **Story origin** | **Summary of the article’s content** | **Main subtheme** | **Other subthemes** | **Media Page** |
| --- | --- | --- | --- | --- | --- | --- | --- |
| 1 | Baby wounded by bullet in mother’s uterus is saved  (Salvan a bebé herido de bala en útero de su mamá) | 2017 | Brasil | Article reporting on a woman and baby in uterus who were wounded by a lost bullet in Brasil. Doctors performed emergency caesarean section (CS), both woman and baby lived. | 2.7 | 1.5 | Azteca Noticias |
| 2 | 13 years of being a mom in vegetative state  (13 años de ser mamá en estado vegetal) | 2014 | Mexico | Article reporting on a 26-year-old Indigenous woman from Oaxaca who has been in vegetal state for 13 years after a CS due to medical negligence, authorities and public health institution have not been held accountable by the justice system. | 3.1 | 3.2  2.1  2.2 | El Universal |
| 3 | Human right’s commission makes recommendation to the public health system for the neurological damage to a baby  (Emite CNDH recomendación a IMSS por daños neurológicos a un bebé) | 2019 | Mexico | Article reporting on Human rights commission making a recommendation to the Instituto Mexicano del Seguro Social (Mexican Institute of Social Security) because of a medical negligence case that led to neurologic damage of a baby | 3.1 |  | El Universal |
| 4 | Post-mortem birth saves baby’s life  (Parto 'post mortem' salva vida a bebé) | 2015 | Mexico | Article reporting on a 14-year-old girl who died in ambulance due to complications of pregnancy. The pregnancy was unknown to paramedics and family, upon the girl’s arrival to the hospital doctors realized she was pregnant and performed a CS. The baby was born premature but healthy. | 1.5 |  | El Universal |
| 5 | Bacteria in vaginal childbirth and breastfeeding benefit babies  (Bacterias en el parto natural y lactancia benefician a bebés) | 2015 | International | Based on the results of a clinical study, the article claims bacteria exposure during vaginal birth and breastfeeding has positive long-term impacts on the health of babies and those who are born through CS have a higher risk of developing allergies. | 1.4 | 1.1  3.3 | El Universal |
| 6 | Doctor accused of medical negligence leading to death  (Acusan muerte por negligencia médica en parto) | 2017 | Mexico | Article reporting on citizens of Indigenous community who organized a protest to ask for the removal of hospital directives due to what they consider negligence against a woman during labour and CS | 3.1 | 3.2 | El Universal |
| 7 | 13-year-old girl dies due to pregnancy complications  (Fallece niña de 13 años por dificultades en embarazo) | 2017 | Mexico | Article reporting on the death of a 13-year-old girl after a CS due to complications (preeclampsia and kidney failure) | 1.5 |  | El Universal |
| 8 | “I was kidnapped in the metro, and a scar saved me”  (“Me secuestraron en el Metro y una cicatriz me salvó”) | 2019 | Mexico | Article reporting on a woman who was abducted and sexually attacked in Mexico City. The woman was released after her captors realized she had a CS scar and told her she was useless because of it | 2.6 | 2.4 | El Universal |
| 9 | What not to eat after a cesarean section  (Qué no comer después de una cesárea) | 2018 | Mexico | Article suggests what foods to avoid during CS recovery such as: soda, coffee, bread, potato, alcohol and high fibre vegetables and legumes | 1.2 |  | El Universal |
| 10 | Half of Mexicans are born through cesarean section  (Nacen por cesárea la mitad de los mexicanos) | 2017 | Mexico | Article tells the story of a woman whose gynaecologist laughed at her wishes of having a vaginal birth during her first prenatal control visit. The gynaecologist told her vaginal birth was outdated and warned her that her bladder would fall. The article states that half of babies in Mexico are born through CS and includes a statement by a medical doctor who claims that almost 9 out of 10 births can be done vaginally | 3.3 | 1.1  1.3  1.4  1.5  2.1 | El Universal |
| 11 | After cesarean section to a girl who was raped, the debate about abortion restarts in Argentina.  (Tras cesárea a niña que fue violada, reavivan debate por aborto en Argentina) | 2019 | Argentina | Article reporting on a 11-year-old girl in Argentina who was pregnant due to sexual violence and received a CS at 5 months instead of a requested early abortion. Activist groups called it torture and the case restarted the debate regarding legal interruption of pregnancy in Argentina. | 2.1 | 1.5  2.6 | El Universal |
| 12 | Cesarean section instead of abortion: the cases of two raped girls that restarted the debate about the legal interruption of pregnancy in Argentina  (Cesárea en vez de aborto: los casos de dos niñas violadas que reavivaron el debate sobre la interrupción legal del embarazo en Argentina) | 2019 | Argentina | Article reporting on two cases of underage girls who were pregnant as a result of sexual violence in Argentina. Both girls were forced to wait to have a CS instead of early abortions. Argentinian law since 1921 has made abortion legal for women who experienced sexual violence. These cases have sparked public debate about the responsibility of the state to guarantee safe abortions. | 2.1 | 1.5  2.6 | El Universal |
| 13 | Midwives, an option against cesarean section.  (Parteras, opción ante la cesárea) | 2017 | Mexico | Article tells the story of 3 traditional midwifes who claim they have never had a case of fetal mortality and have been delivering babies for over 30 years. The midwifes recognize the need for CS in extreme cases but advocate for vaginal birth whenever it is possible. The article also emphasizes the high rates of CS in Mexico and Latin America. | 1.1 | 3.1  1.5 | El Universal |
| 14 | Burns his pregnant sister with gasoline: baby is saved  (Quema con gasolina a su hermana embarazada; salvan a bebé) | 2016 | Mexico | Article reporting on a brother of 25-year-old pregnant woman in Mexico threw gasoline at her and set her on fire, she was taken to the hospital where they performed an emergency CS, she was 8 months pregnant, the baby survived but the woman died of her wounds. The perpetuator of the crime was arrested | 2.7 | 1.5 | El Universal |
| 15 | What you need to know about cesarean section  (Lo que debes saber de la cesárea) | 2016 | Mexico | Article describing CS as a widely used lifesaving surgical procedure that has been overdone according to the World Health Organization. It states that CS is rarely safer than vaginal birth. The article also mentions that: women are awake during CS, babies do not get exposed to bacteria that might be beneficial for their health, it has a longer recovery time than vaginal birth, CS results in scarring, and claims the risk of complications increases with each CS. | 1.3 | 1.1  1.4  1.5  2.5  3.3 | El Universal |
| 16 | Paraguayan 11-year-old will give birth this Thursday  (Menor paraguaya de 11 años dará a luz este jueves) | 2015 | Paraguay | Article reporting on a 11-year-old girl who is pregnant because of rape by her father-in-law. The girl will have a scheduled CS, because abortion is illegal. The criminal has been detained, and the baby's custody will be given to the grandmother. In 2019, 684 girls between 10-14 years of age have given birth in Paraguay. | 2.1 | 2.6 | El Universal |
| 17 | Cesarean section is changing human evolution  (Los partos por cesárea están cambiando la evolución humana) | 2016 | International | Article states that scientist claim CS might be affecting human evolution by preventing the death of women with small pelvis and smaller babies, and that CS could lead to obesity. | 1.5 | 1.3  1.1  1.4 | Muy Interesante México |
| 18 | The method of your birth could affect brain development  (Cómo el método de tu nacimiento podría afectar el desarrollo cerebral) | 2015 | International | Article reporting on an experiment where cs resulted in larger brain cell death in mice before birth compared to vaginal birth | 1.4 | 1.1 | Muy Interesante México |
| 19 | 13-year-old girl and her baby die after emergency cesarean section  (Niña de 13 años y su bebé mueren tras cesárea de urgencia) | 2018 | Argentina | Article reporting on the death of 13-year-old orphan girl and her baby, they both died after an emergency CS, she lived with a 19-year-old male who abused her often. | 2.6 |  | Noticieros Televisa |
| 20 | Cesarean performed on 12-year-old girl, the father of the baby is her relative.  (Realizan cesárea a niña de 12 años, el papá de la bebé es su familiar) | 2019 | Argentina | Article reporting on the case of a 12-year-old girl who delivered a baby through CS, she was pregnant as a result of rape by a relative. | 1.5 | 2.6 | Noticieros Televisa |
| 21 | Enters hospital in Oaxaca for a cesarean section and leaves with burns  (Ingresa a hospital de Oaxaca por una cesárea y sale con quemaduras) | 2015 | Mexico | Article reporting on a woman in Oaxaca who had 3 large burns on her legs due to accidental activation of the electrocautery during her CS. The hospital where she was injured refused to treat her wounds and suggested the woman should seek medical health elsewhere. | 3.1 | 3.2 | Noticieros Televisa |
| 22 | Woman dies in cesarean section and baby is born with scalpel wound  (Muere mujer en operación de cesárea y su bebé nace con herida causada por bisturí) | 2018 | Spain | Article reporting on the case of a Muslim woman who died after an emergency CS due to haemorrhage, the baby survived but was wounded by a scalpel during the procedure. | 3.1 |  | Noticieros Televisa |
| 23 | Doctor performs cesarean section while drunk, mother and baby die  (Médico realiza cesárea estando borracho, muere la madre y su bebé) | 2018 | India | Article reporting the case of a drunk medical doctor who performed a CS on a 22-year-old woman, both mother and child died. The doctor was under investigation for negligence. | 3.1 |  | Noticieros Televisa |
| 24 | Human rights commission makes recommendation to public health system for a negligence case in Chiapas  (CNDH emite una recomendación al IMSS por negligencia en Chiapas) | 2016 | Mexico | Article reporting on recommendations by the Human Rights Commission made to a hospital after a case of medical negligence resulted in the death of a woman and a baby after a CS. The commission also asked the hospital to ensure the woman's partner receives appropriate aid according to the General Law for Victims. | 3.1 |  | Noticieros Televisa |
| 25 | Pregnant woman in Ciudad Juarez is shot, baby is saved  (Balean a embarazada en Ciudad Juárez; salvan a bebé) | 2016 | Mexico | Article reporting on the death of a pregnant Woman who was shot in Ciudad Juarez but medical doctors performed a CS and saved the baby | 1.5 | 2.7 | Noticieros Televisa |
| 26 | Doctors in Chiapas performed a cesarean section during September 7^th^ earthquake  (Médicos en Chiapas realizan cesárea durante sismo del 7 de septiembre) | 2017 | Mexico | Article reporting on the heroism of medical doctors who continued performing an emergency CS on a 16-year-old- girl during a strong earthquake in the state of Chiapas. Girl and baby survived. | 1.5 |  | Noticieros Televisa |
| 27 | Public health system doctors are recognized after performing cesarean section during earthquake in Chiapas  (Reconocen a médicos de IMSS-Prospera que realizaron cesárea durante sismo en Chiapas) | 2017 | Mexico | Article reporting on recognition event by authorities for the medical staff who performed an emergency CS during an earthquake | 1.5 |  | Noticieros Televisa |
| 28 | Children born through cesarean section more likely to suffer obesity or asthma  (Niños nacidos por cesárea: más propensos a padecer obesidad o asma) | 2018 | United Kingdom | Article reporting on study results that suggest women who have had a CS are more likely to have a spontaneous abortion or a stillborn in the future. The article claims that results of the study also suggest that children born through CS are more likely to develop asthma and obesity. | 1.3 | 1.4  3.3 | Noticieros Televisa |
| 29 | Gynaecologists alert about “cesarean section epidemic”  (Ginecólogos alertan sobre una 'epidemia de cesáreas' en el mundo) | 2018 | International | Article reporting on the increasing rates of CS worldwide based on an article published by “The Lancet”. | 3.3 | 1.3  1.4  1.5 | Noticieros Televisa |
| 30 | Mechanism that unleashes autism in childbirth described  (Describen mecanismo que desencadena autismo en el parto) | 2019 | International | Article claims that babies born through CS or problematic births are more likely to develop autism, based on an experiment done in mice | 1.4 |  | Noticieros Televisa |
| 31 | “useless now” woman kidnapped and released for having had a cesarean section  (“Ya no sirve"; secuestran a mujer y la liberan por tener cesárea) | 2019 | Mexico | Article reporting on the case of a woman who was abducted while getting off the metro in Mexico City, she was abused in the car but released when they noticed she had a cs scar, claiming " she was useless now" | 2.6 | 2.4 | Periódico El Debate |
| 32 | They perform cesarean section on 12-year-old girl, the father of the baby is a relative.  (Le hacen cesárea a niña de 12 años, el padre del bebé es un familiar) | 2019 | Argentina | Article reporting on the case of a 12-year-old who had a CS, her pregnancy was the result of sexual abuse by a relative | 1.5 | 2.6 | Periódico El Debate |
| 33 | Baby of abused girl is born and dies after hour of agony  (Nace bebé de niña abusada y muere tras horas de agonía) | 2019 | Argentina | Article reporting on the case of a 12 -year- old girl who had medical doctors perform a CS on her at 23 weeks of pregnancy. The pregnancy was a result of n sexual abuse by her neighbour. The girl's family had asked for an abortion, but doctors refused, afterwards authorities asked the hospital to perform the CS immediately and stated that if the baby survived it will be put up for adoption. The baby had no contact with the girl and died after a few hours | 2.1 | 2.6 | Periódico El Debate |
| 34 | The pain after the death of Agustina, she was 13 years old and she was giving birth  (El dolor tras la muerte de Agustina, tenía 13 años y estaba dando a luz) | 2018 | Argentina | Article reporting on the case of a 13- year-old Indigenous girl who delivered a premature baby through CS. Both the girl and the baby died, she was malnourished and suffering from a respiratory illness, the death revived debate about legal abortion in the country | 2.1 | 2.6 | Periódico El Debate |
| 35 | 13-year-old and her baby die after cesarean section  (Niña de 13 años y su bebé mueren durante cesárea) | 2018 | Argentina | Article reporting on the case of 13-year-old Indigenous girl who an had emergency CS. The girl and the baby died.The girl was malnourished, and an aunt of the girl claimed the girl was an orphan and lived with a 19 year old man who often abused her | 2.6 |  | Periódico El Debate |
| 36 | Natural birth makes better moms than cesarean section  (El parto natural es mejor mamá, que el parto de cesárea) | 2016 | Mexico | Article reporting on the Governor of Nuevo Leon statements about CS. The governor said in a press conference that women should have vaginal births as that will make them "more mothers", and said CS should not be imposed, he also mentioned he would direct public funds to milk banks and delivery rooms. The public reacted negatively to his remarks as they felt he was undermining the motherhood of women who deliver through CS | 2.4 | 2.2 | Periódico El Debate |
| 37 | An elective cesarean section took her life, she left 3 babies behind  (Una cesárea programada le quitó la vida; dejó a tres bebés) | 2018 | Argentina | Article reporting on the case of a young woman who died after a scheduled CS, she complained of pain but was ignored by medical staff, she died 6 hours later. | 3.1 |  | Periódico El Debate |
| 38 | Mom picks up her own intestines after cesarean section  (Mamá recoge sus propios intestinos después de cesárea) | 2019 | United States of America | Article reporting on the case of a woman whose CS wound opened 5 days after the surgery. The woman’s intestines came out, she was taken to the hospital and decided to create awareness of cs risks sharing a photo of the incident on social media | 1.3 | 3.1 | Periódico El Debate |
| 39 | Drunk doctor performs cesarean section, woman and baby die  (Médico realiza cesárea borracho, mueren bebé y madre) | 2018 | India | Article reporting on the case of a doctor in India who performed CS drunk. Both the woman and child died, and there was an investigation to determine the causes of death and assess the doctor’s responsibility | 3.1 |  | Periódico El Debate |
| 40 | It is the most horrible experience, it haunts me  (Es la experiencia más horrible, no deja de perseguirme) | 2018 | Scotland | Article reporting on the case of a doctor in Scotland who refused to perform a CS. The doctor a accidentally decapitated a premature baby while trying to deliver it vaginally. The baby was in an abnormal position and the doctor pulled the baby from its legs. | 3.1 |  | Periódico El Debate |
| 41 | Cesarean section performed on a comatose woman, premature baby is saved in Merida.  (Realizan cesárea a mujer en coma y salvan a bebé prematura en Mérida) | 2019 | Mexico | Article reporting on the case of a premature baby was delivered in the city of Merida through CS. The woman was in a coma due to complications of a brain tumour and had a CS performed on her. The baby was successfully discharged and went home with its father. | 1.5 |  | Periódico El Debate |
| 42 | Brain dead woman gives birth to a baby boy  (Mujer con muerte cerebral da a luz a un varón) | 2019 | Brasil | Article reporting the case of a CS on a woman who brain was dead due to an asthma attack. The woman’s life was prolonged even though she had respiratory decay to give the baby time to grow. | 1.5 |  | Periódico El Debate |
| 43 | Children born through cesarean section are more likely to develop obesity and asthma  (Niños nacidos por cesárea, propensos a obesidad y asma) | 2018 | International | Article reporting on a study that suggests women who deliver through CS have a higher risk of complications in their next pregnancies and births. The article also claims that children born through CS are at higher risk of asthma and obesity. It also states that almost half of all children born in Mexico are delivered by CS | 1.3 | 1.4  1.5 | Periódico El Debate |
| 44 | How to heal cesarean scar after birth  (Cómo curar la cicatriz de la cesárea tras el parto) | 2019 | Mexico | Article providing advice for the healing process after a CS. It suggest good hygiene and to avoid touching the wound, as well as visiting a doctor if any unusual changes occur. | 1.2 | 2.5 | Periódico El Debate |
| 45 | Breast feeding could reduce cesarean section pain  (Amamantar podría reducir el dolor de la cesárea) | 2017 | Spain | Article reporting on a study that suggest that breastfeeding for more than two months could decrease chronic CS pain | 1.2 | 1.3 | Periódico El Debate |
| 46 | Beyonce reveals she had an emergency cesarean section  (Beyonce revela que tuvo cesárea de emergencia cuando dio a luz) | 2018 | United States of America | Article reporting the reveal that a famous singer had an emergency CS. The singer said that after her previous pregnancy she pressured herself to lose weight immediately after birth but was not doing it this time. | 1.5 | 2.5 | Periódico El Debate |
| 47 | Incredible, gives birth to a 6.5 kg baby  (Increíble da a luz a su bebé de ¡6.5 kilos!) | 2017 | United States of America | Article reporting the birth of a 6.5 kg baby through a CS, stating that both the baby and his mother are healthy. | 1.5 |  | Periódico El Debate |
| 48 | Another medical negligence of the public health system, another baby died  (Una negligencia más del IMSS, murió otra bebé) | 2016 | Mexico | Article reporting that the Human Rights Commission was asking for the indemnification of a woman who was refused a CS. The case occurred in the state of Chiapas and the baby died. The commission categorises the case as obstetric violence. | 3.1 | 2.1 | Periódico El Debate |
| 49 | She will receive 200 thousand dollars for being handcuff during childbirth  (Le darán 200 mil dólares por haberla esposado en parto) | 2016 | United States of America | Article reporting that a Mexican Immigrant woman in the USA was handcuffed during labour and CS. The woman filed a lawsuit and wont the case. | 2.2 |  | Periódico El Debate |
| 50 | 11-year-old girl raped by father in law gave birth to her baby  (Niña de 11 años violada por padrastro da a luz a su bebé) | 2015 | Paraguay | Article reporting the case of an 11-year-old girl who had a CS. The pregnancy was a result of sexual abuse by her father-in- law. Initially the family had requested an abortion, but it was denied by the authorities. Their lawyers and human rights organizations are demanding the state to offer the family housing. | 2.1 | 2.6 | Periódico El Debate |
| 51 | It was her uncle who abused Marielos, he was detained today  (Fue su tío quien abusó de Marielos; hoy lo detuvieron) | 2015 | Mexico | Article reporting on the detention of a rapist. The rapist had sexually abused his 13-year-old niece which resulted in pregnancy. The girl had a CS and was diagnosed with HELLP syndrome, and was in a coma for a while. The article reports the girl has been discharged from the hospital. | 2.6 |  | Periódico El Debate |
| 52 | Poorly performed cesarean section in the public health system leaves her 14 years in a coma  (Cesárea mal practicada en el IMSS la deja 14 años en coma) | 2015 | Mexico | Article reporting that an Indigenous woman from Oaxaca has been in a vegetative state for 14 years because of medical negligence during a CS. The family of the woman demands justice. | 3.1 | 2.1 | Periódico El Debate |
| 53 | Childbirth method affects brain development  (Método de nacimiento afecta desarrollo cerebral) | 2019 | International | Article reporting on a study suggesting that CS alters brain development. The study was performed on mice and showed a higher death of brain cells during birth through caesarean section compared to vaginal birth. The study suggests this results could be caused by the lack of bacterial exposure during CS | 1.4 |  | Revista padres e hijos |
| 54 | Cesarean section keeps being abused of, study.  Se sigue abusando de la cesárea, estudio | 2018-2019 | International | Article reporting on a paper by The Lancet which stated that 60% of countries are overusing CS. The article states that when a CS is unnecessary it can increase health risks for both women and babies | 3.3 | 1.5 | Revista padres e hijos |
| 55 | Aftercare of cesarean section scar  Cuidados de la cicatriz de la cesárea | 2018 | Mexico | Article providing tips for a speedy wound recovery after a CS. The tips were mostly focused on good hygiene and healthy eating. | 1.2 | 1.3 | Revista padres e hijos |
| 56 | Know the emotional wound that a cesarean section leaves  (Conoce la herida emocional que deja una cesárea) | 2018-2019 | Mexico | Article that claims women who have CS could develop post-traumatic stress disorder after the procedure | 1.3 |  | Revista padres e hijos |
| 57 | If you have had more than three cesarean sections, careful with your health!  (Si has tenido más de tres cesáreas, ¡cuidado con tu salud!) | 2018-2019 | Mexico | Article reporting on the comments of a medical doctor on CS. The doctor states that multiple CS can lead to scarring of the uterus which can cause placenta accreta and death | 1.3 | 1.5  3.3 | Revista padres e hijos |
| 58 | Benefits of natural childbirth  (Beneficios del parto natural) | 2018-2019 | Mexico | Article detailing the benefits of vaginal birth such as it being the natural way, a speedy recovery, breastfeeding facilitation, low health risks, immediate contact with the baby and positive emotions after birth. | 1.1 | 1.3  1.4 | Revista padres e hijos |
| 59 | Cesarean section, the after.  (Cesárea, el after) | 2019 | Mexico | Article summarizing common issues post CS (such as discomfort and pain, hygiene, post-partum gas) and how to deal with them. It states that recovery takes longer than vaginal birth | 1.2 | 1.3 | Revista padres e hijos |
| 60 | Advice to recover after a cesarean section  (Consejos para recuperarte de una cesárea) | 2019 | Mexico | Article stating that CS is a lifesaving procedure when there is a medical need for it. It also states that private hospitals will push for CS because they can charge patients more. And gives tips for a speedy recovery after a CS: such as good hygiene and avoiding sudden movements. | 1.2 | 1.5 | Revista padres e hijos |
| 61 | Tips to reduce pain after a cesarean section  (Tips para aliviar el dolor por cesárea) | 2019 | Mexico | Article listing suggestions by a medical doctor to relief pain after a CS, such as: pain killers, light walking and cold compresses on the wound. | 1.2 |  | Revista padres e hijos |
| 62 | How to look after your cesarean section wound  (Cómo cuidar tu herida de la césarea) | 2019 | Mexico | Article listing common discomforts after CS (constipation, nausea, pain). The article advices to visit a doctors to address these discomforts and help with recovery. | 1.3 | 1.2 | Revista padres e hijos |
| 63 | Beyonce feared for her life while living through an emergency cesarean section  (Beyoncé temió por su vida al vivir una cesárea de emergencia) | 2018 | United States of America | Article reporting on famous singer who stated she developed pre-clampisa and had to have an emergency CS to deliver her twin children. | 1.5 | 2.5 | Revista padres e hijos |
| 64 | Cuernavaca bar offers “free beer” to women who show their cesarean section scar  (Bar de Cuernavaca ofrece “pomo gratis” a mujeres que muestren su cicatriz por cesárea) | 2018 | Mexico | Article reporting on a bar in the city of Cuernavaca that offered free beer to women who showed her CS scars. Feminist activist protested and started a campaign to censor the promotion labelling the advertising as gender violence. | 2.3 |  | Revista Proceso |
| 65 | With cesarean section and natural birth they inagurate mobile hospital  (Con cesárea y un parto natural estrenan hospital móvil en Juchitán tras sismo) | 2017 | Mexico | Article reporting that a new public mobile health unit was inaugurated by two interventions: a CS and a vaginal birth. | 1.5 |  | Revista Proceso |
| 66 | Congresswoman files a claim against “El Bronco” for saying cesarean section makes less mothers of women  (Denuncia diputada a “El Bronco” por decir que cesárea hace “menos madres” a mujeres) | 2016 | Mexico | Article reporting that a federal congresswoman failed a discrimination claim against Nuevo Leon Governor after he claimed that women are "less mothers" when they have CS. | 2.4 | 2.2 | Revista Proceso |
| 67 | They demand justice to public health system for causing brain damage to Indigenous woman after cesarean section  (Exigen al IMSS justicia por causar daño cerebral a indígena tras cesárea) | 2015 | Mexico | Article reporting that Amnesty International and 2 other NGOs delivered over 800 thousand signatures to IMSS director. Seeking justice for an Indigenous woman who has been in vegetative state for 14 years due to brain damaged caused by medical negligence in a CS | 3.1 | 2.1  2.2 | Revista Proceso |
| 68 | Kylie Jenner refuses to have her baby through cesarean section  (Kylie Jenner se niega a tener a su bebé por cesárea) | 2019 | United States of America | Article reporting that a celebrity is nervous about the possibility of having a CS due to fear of having scars on her body | 2.5 |  | Revista Tu Mexico |
| 69 | Doctor decapitates baby by accident during childbirth  (Doctora decapitó por accidente a bebé durante parto) | 2018 | Scotland | Article reporting on the case of a doctor who accidentally decapitated a baby after deciding not to do a CS, she is under investigation. The baby was in an inadequate position for vaginal birth | 3.1 |  | sdp Noticias |
| 70 | Free beer for cesarean section? The viral promotion for this May 10th  (¿Cerveza gratis por cesárea? La viral promoción para este 10 de mayo) | 2018 | Mexico | Article reporting that a flyer offering free beer for mother's day at a bar if women showed their CS scar went viral. The article claims the flyer was fake. | 2.3 |  | sdp Noticias |
| 71 | Went in for a cesarean section and they mutilated their genitals for being intersexual  (Entró a cesárea y le mutilaron genitales por ser intersexual) | 2016 | England | Article reporting that an activist has started a campaign against genital mutilation after finding out about an intersex woman who had her genitals removed during a CS without her consent | 2.1 |  | sdp Noticias |
| 72 | Gynaecologist forgets cell phone in the womb of patient after cesarean section  (Ginecólogo 'olvida' celular en vientre de paciente a la que le hizo cesárea) | 2015 | Jordan | Article reporting the case of a doctor who allegedly left his cell phone inside a women's abdomen after a CS. | 3.1 |  | sdp Noticias |
| 73 | After cesarean section, doctors forget scissors in women’s womb  (Tras cesárea, doctores olvidan tijeras en vientre de mujer) | 2014 | Panama | Article reporting on a case about doctors who left surgical scissors inside a woman during a CS. The woman lived in poverty and tried to convince doctors something was wrong for 9 months but was ignored, until finally an x-ray confirmed the negligence. | 3.1 |  | sdp Noticias |
| 74 | Cesarean to a raped girl restarts debate about abortion in Argentina  (La cesárea a una niña violada reaviva debate sobre el aborto en Argentina) | 2019 | Argentina | Article reporting on the case of an 11-year-old girl who had a CS. The pregnancy was the result of sexual abuse by her grandmother’s partner. The case revived the debated about abortion, since doctors decided it was too late to interrupt the pregnancy. | 2.1 | 2.6 | Yahoo México |
| 75 | Court blames gynaecologist that attended birth of baby who was decapitated  (Un tribunal culpa a la ginecóloga que atendió el parto de la muerte de un bebé que resultó decapitado) | 2018 | Scotland | Article reporting on the case of a doctor who accidentally decapitated a baby during labour has been found guilty by a court, the baby was in abnormal position and the mother had not dilated enough, a CS should have been performed instead. The article states that it was the first birth for a 30-year-old woman, who claims nobody explained to her what was happening, she was not given any anaesthesia and was not allowed to leave, which she attempted thrice. | 3.1 |  | Yahoo México |
| 76 | Being born through cesarean section could increase the risk of obesity  (Nacer por cesárea aumentaría el riesgo de obesidad) | 2016 | United States of America | Article reporting on a Harvard study which suggest that CS increases the risk of obesity in children by 15% compared to vaginal birth, and that the risk goes up to 64% amongst siblings. The reasons might be the difference in gut bacteria. Expert claimed that although CS is a lifesaving procedure in some cases, risk to the mother and child's health should be considered | 1.4 | 1.5 | Yahoo México |
| 77 | Latin America reigns in a world plagued with unnecessary caesarean sections  (América Latina reina en un mundo plagado de cesáreas innecesarias) | 2015 | Latin America | Article reporting on the high rates of CS in Latin America. Claiming the reasons of the high rates are fear of pain, obstetricians performing CS to benefit their schedules and lack of taxation for the procedure. The articles states that in Latin America 4 out of 10 births are delivered through CS, well above the recommended rate. A woman gives her testimony saying she was too scared of pain therefore she chose a CS and that she would recommend it to her friends. On contrast a medical doctor from Chile explains that some patients think that paying for a procedure would provide better care. CS are mentioned to be 8 times more risky than vaginal births | 3.3 |  | Yahoo México |
| 78 | United States, the country where giving birth can lead to bankruptcy  (Estados Unidos, el país donde dar a luz puede ser una ruina) | 2018 | United States of America | Article claiming that giving birth in the USA can lead to bankruptcy, it tells the story of a family who was ready to welcome triplets. The triplets were delivered prematurely through CS, the family could not pay for the medical bill even though their insurance covered most of it. Although most women have Medicaid which covers the costs of birth, around 56,000 families go bankrupt in the USA after paying for births in USA were CS is up to $20,000 more expensive than vaginal birth. | 1.1 |  | Yahoo México |
| 79 | Cesarean sections have their “effect” on human evolution  (Las cesáreas tienen su 'efecto' en la evolución humana) | 2016 | International | Article reporting that CS are affecting evolution because they are interfering with natural selection | 1.4 | 1.3  1.1 | Yahoo México |
| 80 | Wakes up from a coma thanks to her baby  (Despierta del coma gracias a su bebé) | 2015 | United States of America | Article reporting on the case of a woman who had an emergency CS and wakes from a coma 7 days after the procedure when a nurse placed the baby on the woman’s arms | 1.5 |  | Yahoo México |
