# Supplementary material for: The portrayal and perceptions of cesarean section in Mexican media Facebook pages: a mixed-methods study
Source: Reprod Health. 2022 Feb 22;19:49. doi: 10.1186/s12978-022-01351-8 (PMC8862237; doi:10.1186/s12978-022-01351-8)
Supplement: Supplementary file 2 — Additional file 2: Frequency of all comment codes in comments by article subtheme. [file 12978_2022_1351_MOESM2_ESM.docx]

Appendix S2. This table shows the frequency of all comment codes in comments by Article subtheme

| **Code ID*** | **Article subthemes**  n(%) comments | | | | | | | | | | | | | | |
| --- | --- | --- | --- | --- | --- | --- | --- | --- | --- | --- | --- | --- | --- | --- | --- |
|  | **1.1**  n=66 | **1.2**  n=34 | **1.3**  n=644 | **1.4**  n=611 | **1.5**  n=410 | **2.1**  n=697 | **2.2**** | **2.3** n=194 | **2.4**  n=702 | **2.5**** | **2.6**  n=241 | **2.7**  n=86 | **3.1**  n=1693 | **3.2**** | **3.3**  n=972 |
| **1** | 4 (6.1%) | 2 (5.9%) | 97 (15.1%) | 78 (12.8%) | 22 (5.4%) | 0 | NA | 2 (1.0%) | 21 (3.0%) | NA | 0 | 0 | 17 (1.0%) | NA | 202 (20.8%) |
| **2** | 0 | 9 (26.5%) | 93 (14.4%) | 20 (3.3%) | 7 (1.7%) | 0 | NA | 2 (1.0%) | 4 (0.6%) | NA | 14 (5.8%) | 0 | 2 (0.1%) | NA | 106 (10.9%) |
| **3** | 5 (7.6%) | 10 (29.4%) | 54 (8.4%) | 46 (7.5%) | 8 (2.0%) | 0 | NA | 3 (1.5%) | 13 (1.9%) | NA | 1 (0.4%) | 0 | 6 (0.4%) | NA | 129 (13.3%) |
| **4** | 1 (1.5%) | 2 (5.9%) | 27 (4.2%) | 30 (4.9%) | 3 (0.7%) | 0 | NA | 2 (1.0%) | 10 (1.4%) | NA | 0 | 0 | 3 (0.2%) | NA | 151 (15.5%) |
| **5** | 0 | 0 | 5 (0.8%) | 5 (0.8%) | 149 (36.3) | 83 (11.9) | NA | 118 (60.8) | 241 (34.3%) | NA | 33 (13.7%) | 6 (7.0%) | 10 (0.6%) | NA | 15 (1.5%) |
| **6** | 0 | 0 | 0 | 6 (1.0%) | 84 (20.5%) | 243 (34.9) | NA | 29 (14.9%) | 260 (37.0%) | NA | 62 (25.7%) | 75 (87.2%) | 236 (13.9%) | NA | 2 (0.2%) |
| **7** | 4 (6.1%) | 0 | 1 (0.2%) | 0 | 19 (4.6%) | 15 (2.2%) | NA | 7 (3.6%) | 4 (0.6%) | NA | 2 (0.8%) | 0 | 52 (3.1%) | NA | 2 (0.2%) |
| **8** | 2 (3.0%) | 1 (2.9%) | 212 (32.9%) | 385 (63.0%) | 6 (1.5%) | 0 | NA | 0 | 0 | NA | 0 | 0 | 2 (0.1%) | NA | 28 (2.9%) |
| **9** | 8 (12.1%) | 1 (2.9%) | 37 (5.7%) | 18 (2.9%) | 15 (3.7%) | 8 (1.1%) | NA | 0 | 6 (0.9%) | NA | 4 (1.7%) | 0 | 1,040 (61.4%) | NA | 229 (23.6%) |
| **10** | 2 (3.0%) | 0 | 3 (0.5%) | 0 | 4 (1.0%) | 2 (0.3%) | NA | 0 | 0 | NA | 1 (0.4%) | 0 | 153 (9.0%) | NA | 10 (1.0%) |
| **11** | 2 (3.0%) | 0 | 0 | 0 | 7 (1.7%) | 9 (1.3%) | NA | 1 (0.5%) | 98 (14.0%) | NA | 19 (7.9%) | 5 (5.8%) | 107 (6.3%) | NA | 0 |
| **12** | 0 | 0 | 0 | 0 | 11 (2.7%) | 87 (12.5%) | NA | 1 (0.5%) | 0 | NA | 19 (7.9%) | 0 | 0 | NA | 0 |
| **13** | 0 | 0 | 0 | 0 | 5 (1.2%) | 85 (12.2%) | NA | 0 | 0 | NA | 14 (5.8%) | 0 | 0 | NA | 1 (0.1%) |
| **14** | 0 | 0 | 0 | 0 | 0 | 96 (13.8%) | NA | 0 | 0 | NA | 27 (11.2%) | 0 | 0 | NA | 0 |
| **15** | 0 | 5  (14.7%) | 8 (1.2%) | 8 (1.3%) | 30 (7.3%) | 0 | NA | 0 | 1 (0.1%) | NA | 0 | 0 | 0 | NA | 2 (0.2%) |
| **16** | 0 | 0 | 1 (0.2%) | 3 (0.5%) | 28 (6.8%) | 68 (9.8%) | NA | 22 (11.3%) | 22 (3.1%) | NA | 38 (15.8%) | 0 | 42 (2.5%) | NA | 17 (1.7%) |
| **17** | 18 (27.3%) | 0 | 0 | 3 (0.5%) | 0 | 0 | NA | 0 | 0 | NA | 0 | 0 | 1 (0.1%) | NA | 2 (0.2%) |
| **18** | 20 (30.3%) | 0 | 0 | 0 | 0 | 0 | NA | 0 | 0 | NA | 0 | 0 | 4 (0.2%) | NA | 0 |
| **19** | 0 | 0 | 20 (3.1%) | 7 (1.1%) | 10 (2.4%) | 0 | NA | 6 (3.1%) | 17 (2.4%) | NA | 1 (0.4%) | 0 | 2 (0.1%) | NA | 40 (4.1%) |
| **20** | 0 | 4 (11%) | 86 (13.4%) | 2 (0.3%) | 2 (0.5%) | 1 (0.1%) | NA | 1 (0.5%) | 5 (0.7%) | NA | 6 (2.5%) | 0 | 16 (0.9%) | NA | 36 (3.7%) |

*Code ID: 1.Cesarean section is a lifesaving procedure, 2.Preference for cesarean section, 3.Preference for vaginal birth, 4.Mode of birth is a woman’s choice, 5.Gender violence, 6.Outrage at violence against women, 7.Intersectional discrimination, 8.Disbelief at Information about cesarean section, 9.Discontent at medical practitioners and/or health system 10.Defense of health system and/or medical practitioners 11.Discontent at the government 12.Abortion is a woman’s choice, 13.Against abortion, 14.Abortion only after rape, 15.Breastfeeding, 16.Acknowledgement of discrimination or gender violence, 17.Pro midwives, 18.Against midwives, 19.Women’s worth determined by mode of birth, 20.Negative experience with caesarean section

**There were either no comments or no posts with subthemes 2.2,2.5 and 3.2 as a main theme (See appendix S1).
